# Genome sequence variation in the constricta strain dramatically alters the protein interaction and localization map of *Potato yellow dwarf virus*

**DOI:** 10.1099/jgv.0.000771

**Published:** 2017-06-21

**Authors:** Chanyong Jang, Renyuan Wang, Joseph Wells, Fabian Leon, Mark Farman, John Hammond, Michael M. Goodin

**Affiliations:** ^1^​ Department of Plant Pathology, University of Kentucky, Lexington, KY, USA; ^2^​ USDA-ARS, United States National Arboretum, Beltsville, MD, USA

**Keywords:** rhabdovirus, GFP, *Nicotiana benthamiana*, BiFC, interactome, localization, confocal, nuclear localization

## Abstract

The genome sequence of the constricta strain of *Potato yellow dwarf virus* (CYDV) was determined to be 12 792 nt long and organized into seven ORFs with the gene order 3′-N-X-P-Y-M-G-L-5′, which encodes the nucleocapsid, phospho, movement, matrix, glyco, and RNA-dependent RNA polymerase proteins, respectively, except for X, which is of unknown function. Cloned ORFs for each gene, except L, were used to construct a protein interaction and localization map (PILM) for this virus, which shares greater than 80 % amino acid similarity in all ORFs except X and P with the sanguinolenta strain of this species (SYDV). Protein localization patterns and interactions unique to each viral strain were identified, resulting in strain-specific PILMs. Localization of CYDV and SYDV proteins in virus-infected cells mapped subcellular loci likely to be sites of replication, morphogenesis and movement.

## Abbreviations

BiFC, bimolecular fluorescence complementation; CYDV, Constricta yellow dwarf virus; GST, glutathione-S-transferase; ldr, leader; NLS, nuclear localization signal; PILM, protein interaction and localization map; pI, predicted isoelectric point; PYDV, Potato yellow dwarf virus; SYDV, Sanguinolenta yellow dwarf virus.

## Introduction

Although the coding capacity of viral genomes is low, it is common for each encoded protein to interact with multiple target factors, often located in different subcellular loci [1–3]. Additionally, sequence divergence among viral strains can have profound effects on virulence, symptom development or adaptation to new hosts and vectors [4–8]. As such, determination of the subcellular localization of viral proteins and mapping their interacting partners is fundamental to understanding virus–host interactions [2, 9–12]. Of particular interest in this regard are viruses that are able to replicate in diverse cell types, as proteins encoded by these viruses must contain domains that mediate interaction with factors in evolutionarily divergent hosts.

Rhabdoviruses infect a broad range of hosts, and members of this group includes viruses that infect humans, terrestrial animals/vertebrates, fish, arthropods and plants [9, 13]. Currently, the plant-adapted rhabdoviruses are assigned to two genera, and two more recently described genera, Dichorhavirus and Varicosavirus, contain members with bi-segmented genomes that also infect plants [9, 14, 15]. The genus *Cytorhabdovirus*, for which the type species is *Lettuce necrotic yellows virus*, contains those plant rhabdoviruses that replicate and undergo morphogenesis in the cytoplasm of infected cells [16]. *Potato yellow dwarf virus* (PYDV) is the type species of the genus, *Nucleorhabdovirus*, while *Orchid fleck virus* is the type species of the genus Dichorhavirus. Both of these genera are typified by the nucleotrophic character of member viruses [9, 14, 17, 18].

PYDV was first reported as a highly destructive pathogen of potato (*Solanum tuberosum*), and early research of this virus contributed significantly in the arena of virus–insect interactions [19, 20]. At least seven strains of PYDV have been described at the level of vector-specificity and biological variation in symptom severity [20, 21]. Of these, two strains distinguished by their differential transmission by leafhopper vectors, *Aceratagallia sanguinolenta* and *Agallia constricta*, referred to hereafter as sanguinolenta yellow dwarf virus (SYDV; also called PYDV-New York) and constricta yellow dwarf virus (CYDV; also called PYDV-New Jersey), respectively, became the predominant research strains that served as early models for defining the ultrastructure and cytopathology of plant-adapted rhabdoviruses [22–24] and development of sucrose-gradient centrifugation as an analytical method [25]. Symptom severity of SYDV is greater than CYDV in *Nicotiana benthamiana*, and, in our hands, is easier to purify given its higher titre in that host [18, 26, 27].

The genome of SYDV was characterized previously [18] and, since then, those of several segmented and non-segmented plant rhabdoviruses have been described [2, 9–12]. Collectively, the pattern that has emerged is that the protein interaction and localization maps (PILMs) for each virus are unique [2, 9–12]. Much of this variation is attributable to highly divergent genomic sequences among the viral species. In light of this, we sought to determine if lesser variation in genome sequence could profoundly affect PILMs at the level of viral strains, instead of between viruses. As such, we developed a PILM for the CYDV strain of PYDV and compared it to that of SYDV [18]. It is clear from our studies that even modest changes in sequence variation can affect the topology of PILMs. These studies provide a link between the molecular features of rhabdovirus strains and their differential interactions with host and vector species.

## Results

### Genome sequence of CYDV

**Fig. 1.**
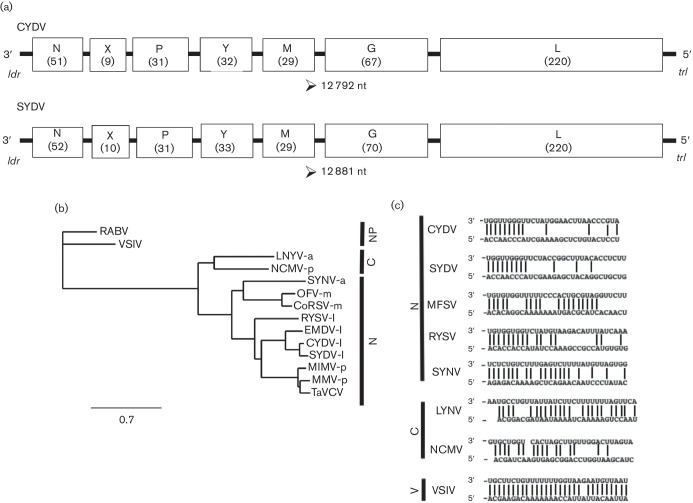
(a) Comparison of the CYDV and SYDV genomes. The 12 792 nt CYDV genome is organized into seven ORFs (open boxes) that are separated by conserved gene junctions and flanked by short leader (ldr) and trailer (trl) sequences, respectively. The predicted size of the encoded protein (in kDa) for each ORF is provided in parentheses. (b) A phylogeny of representative negative-strand RNA viruses was inferred from l-protein amino acid sequences. Viruses infecting a variety of hosts were selected, including those that do not infect plants (NP) as well as plant-adapted species that replicate in nuclei (N) or cytoplasm (C) of infected cells. Vectors for the plant-adapted viruses are provided at the end of the virus abbreviation, namely: a, aphid; l, leafhopper; m, mite; or p, planthopper. Virus names and GenBank accession numbers are listed in Methods. CoRSV, coffee ringspot virus; MMV, *Maize mosaic virus*; TaVCV, *Taro vein chlorosis virus*; MIMV*, Maize Iranian mosaic virus*; OFV, *Orchid fleck virus*; SYDV, *Potato yellow dwarf virus-Sanguinolenta strain;* CYDV*, Potato yellow dwarf virus-Constricta strain*; RYSV*, Rice yellow stunt virus*; SYNV, *Sonchus yellow net virus*; NCMV, *Northern cereal mosaic virus*; LNYV, *Lettuce necrotic yellows virus*; RABV, *Rabies virus*; VSIV, *Vesicular stomatitis virus* – Indiana serotype. All branch points had bootstrap values greater than 0.6. The scale bar indicates the number of aa changes per site. (c) Nucleotide complementarity in the leader (3′) and trailer (5′) regions of selected rhabdoviruses in the *Nucleorhabdovirus* (N), *Cytorhabdovirus* (C) or *Vesiculovirus* (V) genera.

The complete 12 792 nt genome of CYDV, deposited in GenBank as accession KY549567, was determined. The antigenomic sequence has the coding capacity for ORFs, encoding proteins greater than 100 aa each (Fig. 1a). The l gene shares 99 % nucleotide sequence identity with a partial L-gene sequence of a rhabdovirus isolated from Maryland, here identified at CYDV^MD^ (GenBank JQ405264.1). Overall, the genome of CYDV shares 69 % sequence identity with SYDV at the nucleotide level. This variation is distributed more or less evenly across the genome, with the n genes sharing 71 % identity, and the X, P, Y, M, G and L genes sharing 22, 52, 74, 72, 69 and 72 %, respectively. The relationship between CYDV and SYDV is closer if the comparisons are relaxed and similar aa substitutions are considered, i.e. isoleucine and leucine at the same position being considered as functionally equivalent, according to default settings on the blast server. In this scenario, the N, X, P, Y, M, G and L relationships are 83, 43, 73, 88, 83, 88 and 81 % aa similarity, respectively. Interestingly, the CYDV X gene shares greater sequence relatedness (90 % identity) with the cognate protein of *Eggplant mottled dwarf virus* (EMDV). At 52 %, the phosphoproteins of CYDV and SYDV share the lowest aa identity of any cognate pair within the genomes of these viruses.

### Phylogenetics of CYDV based on L-protein sequence comparisons

The phylogenetic relationship of the SYDV strain of PYDV to that of other rhabdoviruses has been established previously [9, 18]. Based on a similar analysis using the primary structure of L proteins, we show that CYDV is most closely related to other leafhopper-transmitted rhabdoviruses, with EMDV being the next most closely related species after SYDV (Fig. 1b) [28]. The aphid-transmitted SYNV, as well as the Brevipalpus mite-transmitted dichorhaviruses, OFV and CoRSV, form clades that are well separated from the leafhopper-transmitted viruses (Fig. 1b). Likewise, the planthopper-transmitted viruses and TaVCV form a separate clade.

### Terminal sequences and gene junctions in the CYDV genome

Regarding SYDV reported previously, the leader and trailer terminal sequences of CYDV have a complete base complementarity over only a very short region, namely the terminal nine bases of the genome (Fig. 1c) [18].

**Fig. 2.**
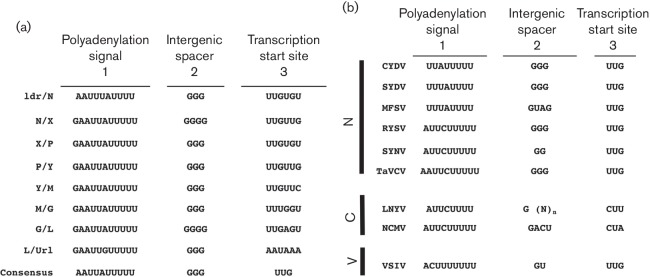
(a) Sequence of each intergenic junction (IGJ) in the CYDV genomic RNA (drawn here in genomic orientation). The IGJs are divided into three regions to denote the [29] poly-adenylation signal, [1] intergenic spacer and [2] transcription start site. The consensus IGJ is provided at the bottom. (b) Consensus IGJ comparisons from rhabdoviruses in the *Nucleorhabdovirus* (N), *Cytorhabdovirus* (C) or *Vesiculovirus* (V) genera. n, variable number of nucleotides.

A conserved gene junction with the consensus 3′-AAUUAUUUUU GGG UUG-5′ (Fig. 2a) was located between each of the ORFs in the CYDV genome, as well as the leader (ldr)/N gene junction. This junction differs from that for SYDV only with respect to the position of the adenine in the poly-U track (Fig. 2b). Overall, the CYDV junctions share a similar tripartite organization with that of other plant-adapted rhabdoviruses, namely: region 1, consisting of a poly-U track that serves as template for poly-adenylation of nascent mRNA transcripts; region 2, a triplet of guanasyl residues; and region 3, the transcriptional start site, consisting of UUG. As is typical for rhabdoviruses, each individual gene junction differs slightly from the consensus sequence. Most notably for CYDV, the intergenic spacer in the N/X and G/L junctions contained an additional guanosine residue (Fig. 2a).

### Predicted features of PYDV proteins

Generally, the predicted sizes of CYDV-encoded proteins are the same as, or slightly smaller, than their SYDV cognates. The N, X and G proteins are approximately 1, 1 and 3 kDa smaller than their SYDV congnates, respectively, whereas the P, Y, M and L proteins are of equivalent sizes for both viruses.

Various protein localization prediction algorithms were used to identify potentially biologically relevant motifs in the CYDV-encoded proteins. A subset of this information is provided in Table 1. Regarding its SYDV cognate, protein localization prediction algorithms failed to identify a nuclear localization signal (NLS) in the CYDV nucleocapsid protein. Furthermore, the primary structure of CYDV-N does not contain the mapped QKRANEEAPPAAQKR bipartite NLS found in SYDV-N [29]. Algorithm-predicted NLSs were identified in the phosphoprotein, matrix protein and polymerase protein.

**Table 1.** Features of PYDV proteins determined by predictive algorithms TM, transmembrane; pI, isoelectric point.

ORF	MW (kD)	TM	pI	Predicted NLS	Putative function	Highest scoring virus/E-value (blast)
1	51	None	7.62	KRTAEDATTQQTKR*	Nucleocapsid (N)	PYDV-N/0.0
2	9	None	3.87	–	Unknown (X)	PYDV-X/4e-04
3	31	None	6.23	PAKSRKL	Phosphoprotein (P)	PYDV-P/2e-103
4	32	None	6.60	–	Movement (Y)	PYDV-Y/3e-161
5	29	None	8.85	KRTVADPFKNLLKRKSE	Matrix protein (M)	PYDV-M/2e-131
6	67	aa 575–597	4.56	–	Glycoprotein (G)	PYDV-G/0.0
7	220	aa 510–529	6.75	KKLPVTNIHPDNLLKKR	Polymerase (L)	PYDV-L/0.0

*This putative NLS was not predicted computationally and is instead the region of the CYDV-N protein corresponding to the mapped NLS in SYDV [29].

Both CYDV and SYDV N proteins have a predicted isoelectric point (pI) of 7.62. The X protein of CYDV has a predicted pI of 3.87, slightly more acidic than the pI 4.5 of its SYDV cognate. Similar to CYDV-X, the P protein at pI 6.23 is slightly more acidic than the 7.72 of its SYDV cognate. The putative movement protein, CYDV-Y, has a pI of 6.6 while that of SYDV is 7.0, both matrix proteins sharing a pI close to 9.0. Both CYDV and SYDV glycoproteins have a pI around 4.6. However, consistent with other proteins, the CYDV-L at pI 6.75 is greater than one log more acidic than its SYDV cognate (pI 7.99).

Although the CYDV-G ORF predicts a smaller protein than its cognate, the relative molecular weight based on the electrophoretic mobility of CYDV-G was reported to be greater than that for SYDV-G (92 kDa versus 85 kDa) [27]. The CYDV-G and SYDV-G proteins are predicted to have seven N-linked glycosylation sites each, and six and nine, respectively, O-linked glycosylation sites. The actual degree of glycosylation has not been mapped physically, and therefore the reason for the difference in electrophoretic mobility of these proteins remains equivocal.

### Localization of CYDV protein fusions in plant cells

**Fig. 3.**
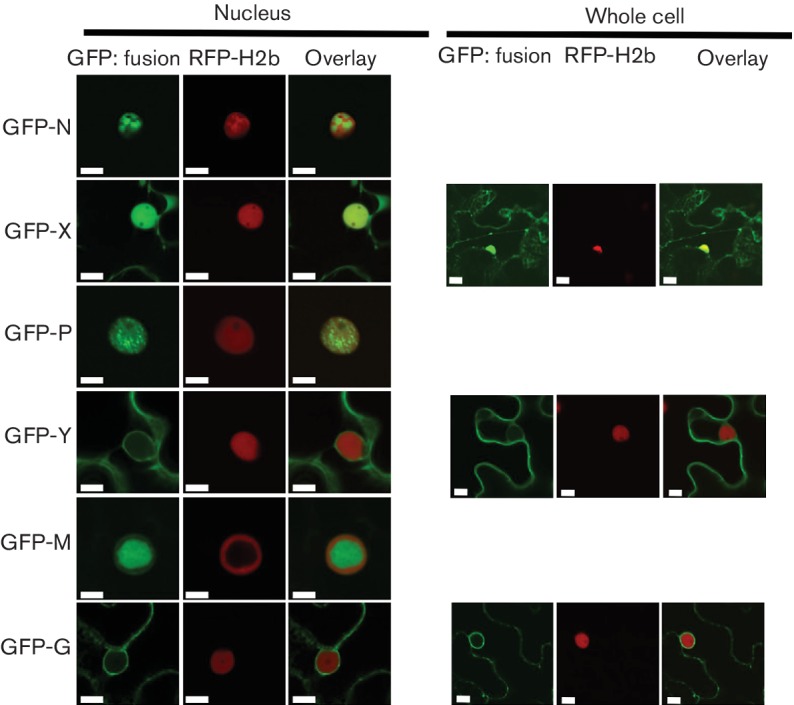
CYDV proteins were expressed using agro-infiltration as amino-terminal fusions to GFP in leaf epidermal cells of *N. benthamiana* plants transgenic for RFP fused to the nuclear marker protein Histone 2B. Particular GFP fusions are listed on the left-hand side of the figure and their corresponding localization in nuclei and whole-cell views is shown to the right and far right, respectively. Whole-cell views are not shown for proteins whose localization was exclusively nuclear. Scale bar, 10 µm.

In order to test whether the sequence variation between SYDV and CYDV influenced protein localization, we determined the subcellular localization patterns for six CYDV proteins *in planta* and compared these data to published results for SYDV [18]. Each of the N, X, P, Y, M and G proteins was expressed as a GFP fusion in transgenic *N. benthamiana*, which expressed RFP fused to histone 2B (Fig. 3). In contrast to GFP:SYDV-N, whose localization was distributed evenly across the nucleoplasm, GFP:CYDV-N localized in sub-nuclear loci with a cross-sectional area of about 2 µm. GFP:CYDV-X distributed throughout the cell, with accumulation in the nucleus. GFP:CYDV-P accumulated in puncta distributed throughout the nucleoplasm, but was excluded from the nucleolus. GFP:CYDV-Y partitioned between the cell periphery and the nuclear envelope, suggesting a membrane association for this protein. Regarding the SYDV matrix protein, the cognate CYDV protein was exclusively nuclear when expressed as a GFP fusion. GFP:CYDV-G associated with endomembranes, with the most easily detectable signal localized on the nuclear envelope.

### Interaction matrix for CYDV proteins

**Fig. 4.**
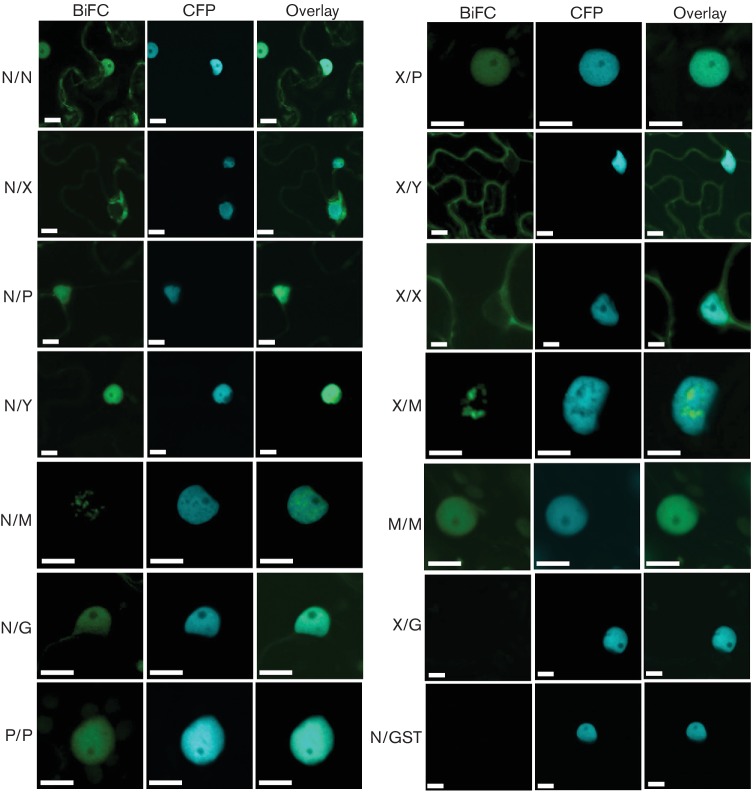
All pairwise combinations for CYDV-encoded proteins, except L, were assayed in bimolecular fluorescence complementation experiments. Specific combinations are listed on the left-hand side of each column of single-plane confocal micrographs that show the location of YFP fluorescence (BiFC) relative to that of the CFP-marked nucleus (CFP). Interaction assays were conducted in leaf epidermal cells of transgenic *N. benthamiana* expressing CFP fused to the nuclear marker protein Histone 2B. The merger of the BiFC and CFP channels is also shown (Overlay). Protein fusions to each half of YFP were tested in all pairwise interactions, of which only a subset is shown here. Glutathione-S-transferase (GST) was used as a non-binding control. The majority of BiFC-negative results are not shown, save those necessary to confirm specificity of binding in the positive assays. Scale bar, 10 µm.

In addition to protein localization studies, we investigated whether the determined sequence divergence between the two viral strains impacted the interaction of CYDV proteins, relative to the interactions observed for SYDV [18]. In order to make direct comparisons, the same type of bimolecular fluorescence complementation (BiFC) assays was used to define the interaction and localization patterns of CYDV proteins (Fig. 4). While all pairwise interactions were tested, in the four protein fusion orientations possible with BiFC, only a subset of the data is reported here. The N, X, P, Y, M and G proteins were tested in all pairwise interactions and against glutathione-S-transferase (GST), which served as a non-binding control (Fig. 4). The L protein was not included in these experiments as we were unable to detect GFP fusions of this protein *in planta* (data not shown). None of the CYDV proteins showed interaction with GST. Positive BiFC interactions were detected for the pairs N/N, N/X, N/P, N/Y, N/M, N/G, P/P, X/P, X/Y, X/X, X/M, and M/M. No other interactions were detected. The X protein did not interact with the G protein.

**Fig. 5.**
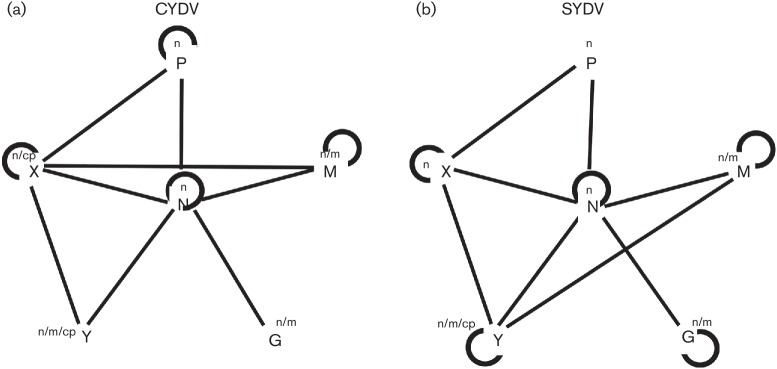
Comparison of integrated protein interaction and localization maps for (a) CYDV and (b) SYDV. Self-interactions are indicated by curved lines. Lines indicate interactions between heterologous proteins. The subcellular localization of GFP-protein fusions is indicated in the superscripts: n, nucleus; n/m, nucleus/membrane; m, membrane; cp, cell periphery.

The resulting BiFC and localization data were integrated into a CYDV PILM, which differs significantly from that of SYDV (Fig. 5). The M/Y, Y/Y and G/G interactions were unique to SYDV, while the P/P and X/M interactions were unique to CYDV.

### Localization of PYDV proteins in virus-infected plant cells

**Fig. 6.**
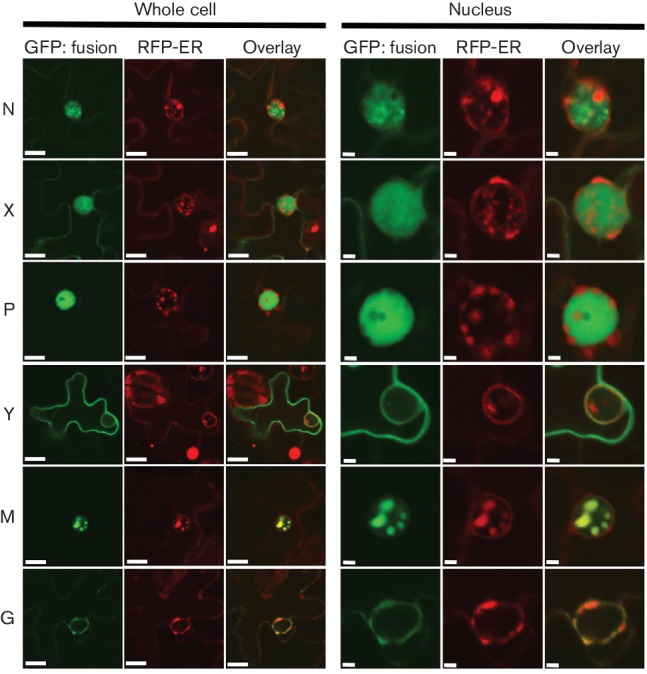
CYDV-encoded proteins were expressed as amino-terminal fusions to GFP in CYDV-infected leaf epidermal cells of *N. benthamiana* plants transgenic for RFP targeted to endomembranes (RFP-ER). Specific CYDV proteins are listed on the left-hand side of the figure and their corresponding localization in whole-cell or nucler views is shown to the left and far left, respectively. Scale bars, 10 µm (whole-cell view) and 2 µm (nuclear view).

**Fig. 7.**
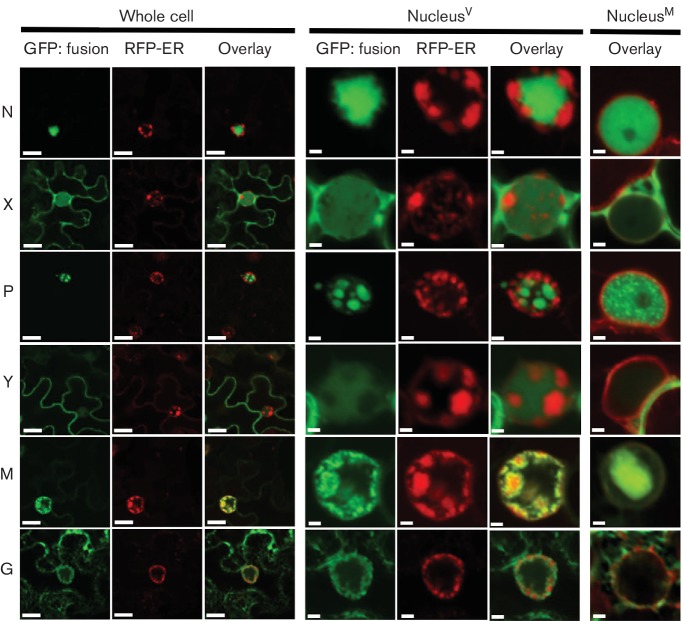
SYDV proteins were expressed as amino-terminal fusions to GFP in SYDV-infected leaf epidermal cells of *N. benthamiana* plants transgenic for RFP targeted to endomembranes (RFP-ER). Specific SYDV proteins are listed on the left-hand side of the figure and their corresponding localization in whole cell or nuclear views is shown to the right and far right, respectively. Scale bars, 10 µm (whole-cell view) and 2 µm (nuclear view). For comparison, the overlay of SYDV protein-GFP fusions in mock-inoculated leaves is shown on the right-hand side.

It has been reported previously that localization patterns of plant-adapted rhabdovirus proteins can differ markedly in the context of infected cells compared to single protein expression in virus-free cells [30]. Given this precedent, we expressed GFP fusions of proteins from both CYDV and SYDV in transgenic *N. benthamiana* plants expressing RFP targeted to the endomembrane system, which provided a facile means to track changes in plant nuclear proteins as well (Figs 6, 7).

GFP:CYDV-N was unevenly distributed throughout the nucleoplasm, while the GFP:CYDV-X and GFP:CYDV-P proteins exhibited a more even distribution throughout the nucleoplasm. In the case of the P protein, the punctate nuclear localization pattern observed when localized in virus-free cells was absent in virus-infected cells. The Y protein showed accumulation on the cell periphery, as well as the nuclear envelope. The GFP:CYDV-M protein co-localized with membranes that accumulated in intranuclear spherules. Regarding its localization pattern in virus-free cells, the GFP:CYDV-G protein accumulated primarily on perinuclear membranes and the nuclear envelope.

In contrast to its cognate protein, GFP:SYDV-N was distributed evenly across the nucleoplasm in virus-infected cells. GFP:SYDV-X protein showed greater partitioning between the nucleus and cytoplasm than GFP:CYDV-X, which was primarily nuclear in the context of infected cells. GFP:SYDV-P was observed on large sub-nuclear foci in virus-infected cells, in a pattern clearly distinguishable from that produced by GFP:CYDV-P. GFP:SYDV-M localized to intranuclear membranes in virus-infected cells, while GFP:SYDV-G was found on endomembranes in the presence or absence of virus.

## Discussion

We have produced a PILM for the CYDV strain of PYDV. The SYDV and CYDV strains represent the closest related plant-adapted rhabdoviruses for which PILMs have been produced. Despite their close sequence relatedness, there are significant contrasts in the protein interaction and localization patterns, which provides insights at the molecular and cellular levels for the contrasting biology of these viruses.

Of particular interest is the difference in CYDV-N of the region spanning the NLS that was mapped in SYDV-N protein (QKRANEEAPPAAQKR) [29]. While the 10 aa spacer is maintained between the paired KR residues essential for nuclear localization, the sequence of the spacer is not conserved, nor are the KR residues flanked by glutamines. Functional mapping will have to verify the KRTAEDATTQQTKR sequence in CYDV-N as being a bona fide NLS. If this is indeed the case, then the charge and sequence variation may explain the marked difference in localization patterns of the PYDV-N proteins, particularly as variation in NLS sequence greatly impacts affinity and isoform selectivity for nuclear import receptors including importin-α, which is the presumed karyopherin for SYDV-N [29, 31]. If this is not the NLS region, then CYDV-N must utilize an entirely different signal to facilitate targeting of this protein to the nucleus. However, given the 71 % sequence identity (83 % similarity) of the CYDV- and SYDV-N proteins, there is no readily identifiable region that might encode an alternative NLS in CYDV-N.

In addition to differences in nuclear localization patterns per se, an intriguing result is the observation that CYDV-Y is targeted to the nuclear envelope, while its SYDV cognate is not. The primary structure of these proteins is 74 % identical and 88 % similar, with the dissimilar residues dispersed over the entire length of the proteins. As such, there is no obvious region in CYDV-Y that readily accounts for targeting of this protein to the nuclear envelope. However, differential interaction with a nuclear transport receptor may account for the differential loclalization pattern [32]. Therefore, the nuclear envelope-targeting region will have to be mapped physically [29]. The difference in partitioning of the Y proteins is maintained in the context of infection, with the SYDV-Y accumulating in the nucleus whereas the CYDV-Y does not. Assuming that the Y protein mediates cell-to-cell transport of PYDV strains, then a differential in the efficiency of transport may, in part, account for the differential symptom severity of these viruses in plants.

While the effect of any single difference in the localization and interactions of PYDV proteins on the general interaction of this virus with its plant host cannot be determined from the present study, collectively it stands to reason that a ‘summation’ of these differences has resulted in vector and plant selectivity. More broadly, it is interesting to note that every plant-adapted rhabdovirus has a unique PILM [2, 10, 11, 18]. All of these PILMs were constructed using BiFC assay in leaf epidermal cells of *N. benthamiana*. Given the uniformity of assay conditions, the differential interactions should be a reflection of the intrinsic properties of each viral protein. BiFC is known to report only very stable interactions, and thus a lack of detecting any particular interaction, e.g. P/P for SYDV or N/N for CoRSV, does not mean that these interactions do not occur but only that they are not stable enough to yield BiFC-positive results. Each protein–protein interaction in the BiFC, and every interaction in general, is governed by a particular dissociation constant (K_D_) [33]. Thus, variation in PILMs, in part, likely represents variation in K_D_ for each viral protein. Extrapolating from the PILMs, it is not uncommon for viral proteins to interact with at least one, and often many more, host cell proteins [1, 2, 34]. Therefore, virus evolution, in particular adaptation to hosts and vectors, must be restricted or permitted according to the efficiency of binding of interaction domains in viral and host proteins [35–37]. Moreover, infection by viruses induces global changes in alternative splicing of host mRNAs [38]. This alternate splicing may alter protein-interaction domains in host factors [39, 40]. Furthermore, interaction with viral proteins can cause dramatic changes in localization of host factors, which may alter their ability to interact with their normal interactors [2]. Coupled with this is the extensive alteration of host cells, particularly nuclear structure, that is evident in CYDV- and SYDV-infected cells. In short, the protein interactome encountered by individual viral proteins is likely to be something quite different from that which exists in the absence of infection.

Taken together, we posit that virus–host cell compatibility is governed, in part, by summation of the efficiency by which viral proteins interact with each other and with host factors. These interactions, in turn, are governed by the K_D_ for each interaction. By corollary, adaptation to new hosts or vectors will be governed by the existence of requisite interaction domains in host factors in new hosts, or sufficiently rapid selection of virus variants from the quasi-species cloud upon entry to a new host or vector. This hypothesis is supported by phylogenetic evidence, which demonstrates that plant-adapted species group according to their insect vector, thus is it likely that insects are the key driver of speciation for this group of viruses [18]. It is intriguing that the X protein of CYDV is more closely related to its cognate protein in EMDV than to SYDV. While there is no firm evidence for recombination between or among these viruses, the solanaceou*s* hosts common to both may have provided such an opportunity [41]. Thus, variation in PILMs is likely expected given the diverse host range that can be collectively infected by the plant-adapted rhabdoviruses for which PILMs have been generated. Furthermore, within a single-host species, e.g. *N. benthamiana*, plant-adapted rhabdoviruses exhibit a wide range of pathogenicity, with some viruses expressing a recovery phenotype (SYNV) [26], taking exceptionally long (weeks) to establish infections (PYDV) [26], or requiring plants to be maintained at elevated temperatures in order to establish systemic infections (CoRSV) [10].

Mechanistic investigation of the hypotheses above will require expansion of the availability of recombinant viral systems [42, 43] and detailed biochemical characterization of rhabdoviral protein complexes, with particular attention paid to the determination of K_D_s for interactions contributing to PILMs, as well as a broader characterization of host factors that interact with plant rhabdoviral proteins [2]. However, the availability of a significant number of PILMs raises intriguing questions about their underlying molecular basis, which have implications for understanding the evolutionary trajectories of these viruses.

## Methods

### Virus maintenance and purification

All plants, including transgenic *N. benthamiana* lines expressing autofluorescent proteins fused to histone 2B, a nuclear marker, or RFP-HDEL (endomembrane marker), were maintained in the greenhouse under conditions reported previously [30]. *Potato yellow dwarf virus* strain CYDV (American Type Culture Collection accession PV-233) was maintained by serial passage in *N. benthamiana* and *N. rustica* plants housed in insect-proof cages. As reported for SYDV, CYDV was purified on sucrose density gradients, as described previously [26]. Field isolates of CYDV were collected from infected tomato (*Solanum lycopersicum*) in 2010, and black nightshade (*Solanum nigrum*) and pepper plants (*Capsicum annum*) in the fall of 2016 in Beltsville, MD (Hammond, unpublished data). This isolate will hereafter be referred to as CYDV^MD^.

### Isolation of total RNA, RT-PCR

Total RNA was extracted from plant tissues using the Qiagen RNeasy Plant minikit (Qiagen). Except where noted, first-strand cDNA synthesis and PCRs were carried out using Superscript reverse transcriptase IV (Thermo Fisher Scientific) and Phusion high-fidelity DNA polymerase (Finnzymes), respectively.

### ION Torrent sequencing

The genomic sequence of CYDV was determined using the same ION Torrent sequencing pipeline utilized for determination of the CoRSV genome [10]. All library construction and sequencing steps were performed by staff of the Advanced Genetic Technology Center (University of Kentucky). Poly(A)^+^-RNA was purified from total RNA isolated from CYDV-infected leaves of *N. rustica* at 30 days post inoculation using a Dynabeads mRNA Purification Kit according to the manufacturer’s instructions. Template cDNA was prepared using an IonPGM Template OT (One-Touch) 200 Kit. Sequencing was performed with an Ion PGM Sequencing 200 Kit and the Ion 316 chip. Contigs were assembled from the high-quality read data using the Trinity assembler package [44].

### RACE

3′- and 5′-RACE were performed with the BD-SMART RACE cDNA Amplification kit according to the manufacturer’s instructions (Thermo-Scientific). For these analyses, cDNA was synthesized by MMLV reverse transcriptase, and PCRs were conducted with Advantage-II DNA polymerase (Clontech).

### DNA sequence analysis

Homology searches were used to compare CYDV sequences to the genomes of other rhabdoviruses using various blast tools provided on the National Center for Biotechnology Information (NCBI) server. ORFs were identified using the ORF finder search tool [45]. The primary structures of proteins encoded by CYDV were analysed using a variety of algorithms provided by the Expasy proteomics server [46], including Compute pI/MW [47], psort for prediction of protein localization [48], SignalP for prediction of signal peptide cleavage sites [49] and NetNGlyc for prediction of N-glycosylation sites [50].

### Phylogenetic analysis

All L-protein sequences used in the sequence alignment study were obtained from data deposited in the NCBI database. In addition to that for CYDV, L-gene sequences utilized in phylogenetic analyses include the following: Coffee ringspot virus – Lavras strain (CoRSV; KF812526), Eggplant mottled dwarf virus (EMDV; NC_025389, Lettuce necrotic yellows virus (LNYV; AJ867584), Maize mosaic virus (MMV; AY618418.1), Sonchus yellow net virus (SYNV; L32603.1), Maize fine streak virus (MFSV; AY618417.1), Potato yellow dwarf virus (PYDV; NC_016136.1), Maize Iranian mosaic virus (MIMV; DQ186554), Northern cereal mosaic virus (NCMV; NC_002251.1), Orchid fleck virus (OFV; NC_009609), Rice yellow stunt virus (RYSV; NC_003746.1), Taro vein chlorosis virus (TaVCV; NC_006942.1) and Vesicular stomatitis Indiana virus (VSIV; NC_001560.1).

Sequence alignment and phylogenetic trees, generated using the neighbour-joining method with a bootstrap test with 1000 replicates, were conducted using the Phylogeny.fr suite of online tools, as described previously [51, 52].

A partial sequence of the L gene from CYDV^MD^ (isolated from tomato) was recovered by PCR using generic plant rhabdovirus primers [53]. This sequence had a 99 % nucleotide sequence identity to CYDV, and was deposited in GenBank as accession (no. JQ405264).

### Protein expression in plant cells

Sequence-validated clones in vector pDONR221 (Invitrogen) of all CYDV ORFs, except L, were recombined into the appropriate binary vectors for localization of fluorescent protein fusions in plant cells [30, 54]. Tests for protein interactions were conducted using BiFC assays as described previously [10–12, 55]. Importantly, the conversion of the pSAT-based vectors to allow Gateway recombination-based cloning entirely eliminated the high background when ‘empty’ vectors expressing the two halves of YFP were co-expressed. As such, we have determined that false positives are less likely to occur when using the pSITE-BiFC vectors [2, 55]. Therefore, the vectors employed in this study were pSITE-2CA (GFP fusions) and localization experiments, and the pSITE-BiFC-nEYFP and pSITE-BiFC-cEYFP vectors for BiFC assays. Recombinant vectors were transformed into *Agrobacterium tumefaciens* strain LBA4404. Agroinfiltration for expression of protein fusions in plant cells was conducted essentially as described previously [30].

### Laser scanning confocal microscopy

Microscopy for this study was conducted using an Olympus FV1000 laser-scanning confocal microscope as described previously [30].
